# Assessment of Programmed Cell Death Ligand-1 Expression in Oral Potentially Malignant Disorders and Tumor-Free Surgical Margins of Oral Squamous Cell Carcinoma: Protocol for a Retrospective Cross-Sectional Study

**DOI:** 10.2196/82568

**Published:** 2026-04-16

**Authors:** Archana Sonone, Alka Hande, Swati Patil, Aayushi Pakhale, Preethi Sharma

**Affiliations:** 1 Department of Oral Pathology and Microbiology, Sharad Pawar Dental College and Hospital, Datta Meghe Institute of Higher Education and Research, Sawangi (Meghe), Wardha, Maharashtra, 442004, India, 91 9145229353

**Keywords:** oral squamous cell carcinoma, oral potentially malignant disorders, surgical margins, programmed cell death ligand-1, PD-L1, immunohistochemistry, prognosis, survival

## Abstract

**Background:**

Oral squamous cell carcinoma (OSCC) is a highly prevalent and aggressive malignancy of the oral cavity, frequently preceded by oral potentially malignant disorder (OPMD). Despite therapeutic advances, survival rates remain unsatisfactory, primarily due to late diagnosis, recurrence, and molecular alterations in histologically tumor-free surgical margins. Programmed cell death ligand-1 (PD-L1), an immune checkpoint molecule, contributes to tumor immune evasion and has been implicated in cancer progression. Its expression in OPMDs and OSCC surgical margins may serve as an early indicator of malignant transformation and recurrence risk.

**Objective:**

This study aims to assess PD-L1 expression in OPMDs and histologically negative surgical margins of OSCC and evaluate their association with 3-year survival outcomes.

**Methods:**

This retrospective cross-sectional study will be conducted over 12 months at a tertiary care hospital in Sawangi Meghe, Wardha, India. Archived formalin-fixed, paraffin-embedded samples of OPMDs and tumor-free surgical margins from OSCC cases (2018‐2020) will be retrieved. Immunohistochemistry for PD-L1 will be performed using the SP263 clone, and expression will be evaluated using the combined positive score. Demographic, clinical, and survival data will be collected from patient records. Statistical analysis will determine correlations among PD-L1 expression, clinicopathological variables, and 3-year survival.

**Results:**

The study is expected to provide insights into the role of PD-L1 as a biomarker for early detection, prognostication, and risk stratification in patients with OPMDs and OSCC. Data collection and immunohistochemical analysis have not yet commenced at the time of submission.

**Conclusions:**

By evaluating PD-L1 expression in premalignant lesions and histologically negative margins, this study aims to identify molecular predictors of OSCC progression and survival. The findings may help establish PD-L1 as a prognostic biomarker and support its integration into precision oncology and immunotherapy strategies.

## Introduction

### Background

The most prevalent cancer that affects the oral and maxillofacial areas is oral squamous cell carcinoma (OSCC), which still has a major negative impact on global health. Head and neck cancers rank eighth worldwide according to GLOBOCAN 2020, with OSCC alone responsible for over 440,000 annual deaths and approximately 880,000 new cases [1,2]. Oral potentially malignant disorders (OPMDs), which impact approximately 2.5% of the general population, are the cause of a significant percentage of OSCCs [3]. Oral leukoplakia, oral submucous fibrosis, and oral lichen planus are the most common OPMDs; they all have varying but noteworthy potential for malignant transformation [3].

Overall 5-year survival rates for OSCC have remained at roughly 50%, indicating that the prognosis has not improved much in recent decades despite advancements in surgery, radiotherapy, and chemotherapy [2]. Although most patients present at advanced stages, the prognosis is better in early, well-differentiated tumors. Local recurrence is frequent even in cases with histologically tumor-free margins, which suggests that conventional histopathology frequently misses minimal residual cancer cells [4,5].

Immunohistochemistry (IHC) has become a useful supplementary technique for identifying molecular alterations in tissues that seem histologically normal to get around this restriction. IHC analysis of negative surgical margins may be useful in identifying patients who are more likely to experience a recurrence because genetic and molecular changes occur before phenotypic transformation [5]. Nevertheless, the effectiveness of these methods mainly depends on trustworthy biomarkers that can differentiate cancerous from healthy epithelial cells and offer prognostic information [6].

The programmed cell death ligand-1 (PD-L1) is one of the markers that has been studied the most in recent years. The type I transmembrane protein PD-L1, also referred to as CD274 or B7-H1, is a member of the B7 family and consists of an intracellular cytoplasmic domain, a transmembrane segment, and extracellular immunoglobulin V– and immunoglobulin C–like domains [7,8]. By binding to the programmed cell death receptor-1 (PD-1) expressed on T cells, PD-L1 plays a crucial role in immune checkpoint regulation under physiological conditions. This inhibits T-cell proliferation, cytokine production, and cytotoxic activity [8,9]. The prevention of autoimmunity and preservation of immunological tolerance depend on this pathway. Nevertheless, abnormal PD-L1 expression in the tumor microenvironment allows cancerous cells to elude immune monitoring, which advances the course of the disease [10,11].

It is still unclear how PD-L1 functions in the early phases of oral carcinogenesis and in predicting OSCC recurrence despite the fact that it is now a recognized biomarker for directing immune checkpoint inhibitor treatments in a number of cancers [9,12]. According to recent data, PD-L1 may be expressed in both tumor-free margins of OSCC and OPMDs, suggesting that it plays a role in residual disease as well as malignant transformation [3,10,12]. The clinical significance of this pathway has also been highlighted by clinical trials using immune checkpoint inhibitors such as pembrolizumab and nivolumab, which have shown antitumor efficacy in recurrent and metastatic head and neck squamous cell carcinoma (HNSCC) [11,13].

PD-L1 expression is characterized by significant biological and technical heterogeneity, influenced by tumor microenvironment, inflammatory infiltrate, assay, platforms, antibody clone, and scoring algorithms. In HNSCC, variability in PD-L1 expression across tumor compartments and disease stages has been widely reported, complicating the interpretation of PD-L1 as a stand-alone biomarker by clinicians and researchers. Furthermore, discordance among primary tumor, surgical margins, and premalignant lesions highlights the dynamic nature of PD-L1 expression during carcinogenesis. Evidence from other epithelial malignancies, including lung and breast cancer, underscores the prognostic relevance of PD-L1 while simultaneously emphasizing challenges related to cutoff selections, interobserver variability, and the temporal instability of expression. Recognizing these nuances is essential when evaluating PD-L1 as a screening or prognostic biomarker in OPMDs and OSCC.

Several studies have demonstrated that PD-L1 expression is not restricted to advanced disease but may be detectable during early oral carcinogenesis. Yoshida et al [14] reported significant PD-L1 expression in tongue squamous cell carcinoma, suggesting its involvement in tumor development and progression. Furthermore, Schneider et al [15] demonstrated that PD-1 and PD-L1 expression in primary HNSCC and corresponding lymph node metastases had a significant impact on clinical outcomes. These findings support the hypothesis that PD-L1 expression may serve as an early molecular indicator of aggressive biological behavior in oral malignancies [14,15].

Biomarker-driven strategies that support risk stratification, early detection, and prognosis prediction are desperately needed, especially in light of the increasing prevalence of OPMDs and OSCC. In light of this, this study intends to assess PD-L1 expression in OSCC tumor-free surgical margins and OPMDs, as well as investigate its relationship to clinicopathological characteristics and patient survival. This study aims to produce evidence that might support the use of PD-L1 as a prognostic marker in the clinical management of OSCC by examining the continuum from premalignant lesions to recurrence.

### Study Objectives

#### Primary Objective

The primary objective is to compare PD-L1 combined positive score (CPS) expression between OPMDs and tumor-free surgical margins of OSCC.

#### Secondary Objectives

The secondary objectives are to (1) evaluate PD-L1 CPS expression in the normal oral mucosa as a reference group and (2) assess the association between PD-L1 CPS expression in tumor-free surgical margins and 3-year overall survival (OS) and disease-free survival (DFS) in patients with OSCC.

### Study Outcomes

#### Primary Outcome

The primary outcome is PD-L1 expression, measured using the CPS in OPMDs and tumor-free surgical margins of OSCC.

#### Secondary Outcomes

The secondary outcomes are as follows: (1) PD-L1 CPS expression in the normal oral mucosa, (2) 3-year OS and DFS in patients with OSCC, and (3) the correlation between PD-L1 CPS expression in tumor-free margins and survival outcomes.

## Methods

### Overview

This retrospective analysis will be conducted between June 2025 and June 2026 using archival samples collected from 2018 to 2020. All analyses will be performed on existing clinical and survival data available in patient records. This is a retrospective study conducted in the Department of Oral Pathology and Microbiology at Sharad Pawar Dental College and Hospital, Datta Meghe Institute of Higher Education and Research (Deemed to be University), located in Sawangi (M), Wardha, Maharashtra, India. Archived formalin-fixed, paraffin-embedded tissue samples and the corresponding clinical records of patients diagnosed with OPMD and surgically treated OSCC between January 2018 and December 2020 will be retrieved from departmental archives. All clinical outcomes, including survival status, recurrence, and follow-up details are based on retrospective review of medical records, with a minimum follow-up duration of 3 years where available. The study will be conducted over a 12-month period (June 2025 to June 2026), which will include retrieval of archival samples, immunohistochemical analysis, data extraction, statistical analysis, and manuscript preparation. No prospective data collection or follow-up is planned. The eligibility criteria, including detailed inclusion and exclusion parameters, are summarized in Table 1. This study protocol has been prepared in accordance with the STROBE (Strengthening the Reporting of Observational Studies in Epidemiology) guidelines for retrospective studies.

**Table 1.** Inclusion and exclusion criteria.

Category	Description
Inclusion criteria	Patients with a primary diagnosis of OPMDs^a^ or OSCC^b^, tumor sites restricted to the oral cavity, and histologically tumor-free surgical margins
Exclusion criteria	Recurrent tumors, patients lost to follow-up, variants of squamous cell carcinoma, and patients with histologically positive surgical margins

a OPMD: oral potentially malignant disorder.

b OSCC: oral squamous cell carcinoma.

The sample will be divided into three groups: (1) histologically diagnosed OPMD cases (20 samples; group A), (2) histologically tumor-free surgical margins of OSCC cases (20 samples; group B), and (3) normal oral mucosa (20 samples; group C).

The departmental archives will be searched for tumor-free surgical margins of OSCC cases and histopathologically diagnosed instances of OPMDs. The samples will be taken from the 2018 to 2020 time frame in the archives. At the beginning of the trial, the patients’ current state of survival will be recorded. Each patient will have an additional 3-year follow-up.

### Parameters That Will Be Studied

Both histopathological and clinical outcomes will be evaluated. They will be classified into 2 major groups: OPMDs and OSCC surgical margin cases.

#### Histopathological Parameters

For OSCC tumor-free surgical margins, the following parameters will be evaluated:

- Margin status (distance from tumor in millimeters; clear vs close vs involved)
- Presence of dysplasia (mild, moderate, or severe)
- Lymphovascular invasion
- Perineural invasion
- Pattern of invasion (cohesive or noncohesive)
- Histological grade of the primary tumor (well, moderately, or poorly differentiated)
- Depth of invasion
- Extranodal extension if lymph node metastasis is present

For OPMDs, the following parameters will be evaluated:

- Type of lesion (eg, leukoplakia, oral submucous fibrosis, erythroplakia, or lichen planus)
- Degree of epithelial dysplasia (World Health Organization grading: none, mild, moderate, severe, or carcinoma in situ)
- Architectural and cytological alterations

The assessment of both OS and DFS or malignant transformation–free survival (for OPMD) will be conducted based on the data available.

#### Clinical Parameters

The following clinical parameters will be evaluated:

- Age and gender
- Risk habits (tobacco, betel quid, or alcohol use)
- Site of lesion (eg, tongue, buccal mucosa, or floor of mouth)
- Date of diagnosis or surgery
- Date of last follow-up or death

### Survival Analysis Plan

#### Overview

Kaplan-Meier survival curves will be used to visualize OS, DFS, or malignant transformation–free survival. The log-rank test will be used to compare survival between groups (eg, positive vs negative margins). The Cox proportional hazard model will be used to identify independent predictors of survival (eg, margin status, depth of invasion, and dysplasia grade).

#### IHC Analysis

The assessment of PD-L1 antigen will be performed via IHC. The Universal Immuno-enzyme Polymer method will be used for this assessment. After deparaffinization with xylene, tissue sections will be immersed into grades of alcohol for rehydration. The microwave heat antigen retrieval technique will be used. Under this technique, sections will be heated for 10 minutes in 0.01 M sodium citrate buffer (pH 6.0). They will then be cooled at room temperature for approximately 20 minutes. This procedure will be carried out repeatedly until enough antigen is retrieved. After this, sections will be washed with the help of phosphate-buffered saline (PBS) and treated with 3% hydrogen peroxide in methanol for 30 minutes to block endogenous peroxidase activity. For nonspecific binding of the antigen, the section will be rinsed with PBS 3 times. The primary PD-L1 antibody (rabbit monoclonal anti–PD-L1 antibody [SP263 clone; Roche Diagnostics]) will then be applied to the tissue section and incubated for 90 minutes at room temperature in a humidifying chamber. Again, the section will be washed for 3 to 10 minutes with PBS and polymer antimouse antibody conjugated with horseradish peroxidase for 30 minutes. The antigen-antibody reaction will be observed using 3,3’-diaminobenzidine chromogen in a buffer solution. Mayer’s hematoxylin counterstaining will be used. PD-L1 immunoexpression will be assessed by 2 independent observers in a blinded manner. Interobserver variability will be evaluated using appropriate statistical methods such as the Cohen κ for categorical data or the intraclass correlation coefficient for continuous data.

PD-L1 expression will be assessed using the CPS under light microscopy. Two pathologists will conduct the evaluation blinded without access to the clinical details.

The CPS is calculated by dividing the number of PD-L1 immunoexpression-positive cells, including cells of the epithelium, macrophages, and lymphocytes, by the total number of viable neoplastic cells and then multiplying the result by 100 [13], as follows: CPS = (number of PD-L1–staining cells [tumor cells, lymphocytes, and macrophages]/total number of viable tumor cells) × 100.

The CPS was selected as it is the most clinically validated scoring algorithm for PD-L1 assessment across multiple tumor types. Variability in PD-L1 interpretation due to differences in antibody clones, platforms, and scoring systems has been widely documented, highlighting the need for standardized evaluation methods [16].

### Statistical Plan

One-way ANOVA or Kruskal-Wallis tests will be used to compare the CPS across the 3 study groups, followed by post hoc pairwise comparisons where appropriate. To assess the correlation between CPS in surgical margins and 3-year survival, the Spearman rank correlation or logistic regression may be used. If survival data are available as time to event, Kaplan-Meier survival analysis with the log-rank test is recommended.

### Ethical Considerations

Institutional Ethics Committee approval has been obtained from Datta Meghe Institute of Higher Education and Research (DMIHER(DU)/IEC/2025/42). Every participant in the study has given their informed consent. Participant confidentiality will be maintained by anonymizing data and removing identifiable information. Data will be securely stored and accessed only by the research team. No monetary or nonmonetary compensation will be provided.

## Results

This manuscript describes the protocol for a retrospective cross-sectional study. Data collection and immunohistochemical analysis are scheduled to commence in June 2025 and continue until June 2026. Results will be reported in a subsequent publication upon completion of the analysis, expected by late 2026 or early 2027.

The planned analyses include descriptive statistics of baseline clinical and demographic characteristics; assessment of PD-L1 expression in OPMDs, tumor-free surgical margins in OSCC, and normal oral mucosa; comparative analyses of PD-L1 expression between study groups; and Kaplan-Meier survival analysis and Cox proportional hazard regression to evaluate the association between PD-L1 expression and 3-year OS and DFS. Institutional ethics committee approval was obtained in January 2025 (DMIHER(DU)/IEC/2025/42). No external funding was received. Archival retrieval, IHC staining, and data extraction are planned from June 2025 to June 2026. As this is a retrospective study using cases from 2018 to 2020, no prospective recruitment is required. Data analysis is scheduled for mid-2026, and results are expected to be published by late 2026 or early 2027.

Final results will be reported in accordance with the study objectives upon completion of the analysis.

## Discussion

### Expected Findings

OSCC remains a major global health burden, with over 377,000 new cases and 177,000 deaths annually, ranking among the leading causes of cancer-related mortality worldwide [2]. A significant proportion of OSCCs arise from OPMDs, highlighting the importance of early biomarkers that can predict malignant transformation and guide patient management [3]. Despite advancements in surgical and adjuvant therapies, disease recurrence and poor survival remain key challenges, particularly due to residual tumor cells in histologically tumor-free surgical margins [4,6].

PD-L1 has emerged as a promising immune biomarker across multiple malignancies owing to its central role in tumor immune evasion [8,9]. Its overexpression has been reported in OPMDs as well as in invasive OSCC, suggesting a potential role in both early carcinogenesis and disease progression [3,10,12]. Studies such as those by Dave et al [10] and Pachpande et al [3] demonstrate that PD-L1 immunopositivity increases progressively from OPMDs to frank carcinoma, reinforcing its utility as a screening marker. A recent systematic review further supports the prevalence of PD-L1 expression in precancerous lesions of the head and neck, although with significant heterogeneity in cutoff values and detection methods [12].

In the context of surgical margins, molecular and immunohistochemical analyses provide deeper insights than conventional histopathology alone [4,6]. While histologically clear margins are the current standard of care, residual molecular alterations can still be predisposed to recurrence. Braakhuis et al [4] emphasized that molecular mapping of margins reveals “field cancerization” phenomena, and Ranka et al [6] specifically highlighted PD-L1 as one of the immunohistochemical markers with prognostic relevance. Thus, assessing PD-L1 in tumor-free surgical margins may help identify patients at higher risk of recurrence despite negative histopathology.

PD-L1 expression has also been linked to patient prognosis and survival outcomes in other cancers, including lung [17] and breast cancers [18], as well as in advanced solid tumors treated with immune checkpoint inhibitors [11]. In head and neck cancer, immunotherapy with anti–PD-1 and PD-L1 agents (eg, nivolumab and pembrolizumab) has shown promising activity, particularly in PD-L1–positive patients [13]. These findings underscore the translational value of PD-L1 as both a prognostic and predictive biomarker in OSCC [19].

PD-L1 expression has also been shown to correlate with key molecular and behavioral risk factors. Tojyo et al [20] and Ahmadi et al [21] reported significant associations between PD-L1 expression and p53 status, smoking habits, and sex, suggesting that PD-L1 upregulation may reflect underlying genomic instability and carcinogen exposure. These associations further support the biological plausibility of PD-L1 as a prognostic and risk stratification biomarker in OSCC [20,21].

The clinical relevance of PD-L1 expression is further supported by therapeutic response data. Tang et al [22] reported a favorable response to nivolumab combined with radiotherapy and nimotuzumab in a metastatic patient with OSCC with strong PD-L1 expression, highlighting the predictive potential of this biomarker. Additionally, activation of the PD-1 and PD-L1 pathway has been linked to cancer-related pain, underscoring its broader biological and clinical significance beyond immune evasion alone [22,23].

At the same time, interpretation of PD-L1 expression remains challenging due to considerable biological and technical variability. PD-1 expression is influenced by factors such as the tumor microenvironment, inflammatory infiltrate, assay platforms, antibody clones, and scoring methods. In HNSCC, wide variations in PD-L1 expression across tumor sites and disease stage have been reported, limiting its use as a stand-alone biomarker. Differences in expression among primary tumors, tumor-free surgical margins, and premalignant lesions further indicate that PD-L1 expression may change during the process of carcinogenesis. Evidence from other epithelial malignancies such as lung and breast cancers similarly supports the prognostic relevance of PD-L1 while emphasizing the persistence of challenges related to cutoff selection, interobserver variability, and the temporal instability of expression. Recognizing these nuances is critical when evaluating PD-L1 as a potential screening or prognostic biomarker in OPMD and OSCC.

Given this evidence, our study aims to comprehensively evaluate PD-L1 expression through the CPS in OPMDs and OSCC surgical margins and correlate its expression with 3-year survival outcomes. By integrating biomarker assessment with clinical follow-up, this study will contribute to bridging the gap between molecular diagnostics and prognostic modeling in OSCC.

### Study Limitations

This study has certain limitations inherent to its retrospective design. Although the sample size is sufficient to address the primary objective, the relatively small number of cases per group may limit the detection of subtle associations and restrict the feasibility of detailed subgroup analyses. Additionally, survival analyses are dependent on the completeness and accuracy of archival follow-up records. These limitations will be carefully considered during the interpretation of the results and highlight the need for larger, prospective studies to validate the findings.

## Abbreviations

CPS: combined positive score
DFS: disease-free survival
HNSCC: head and neck squamous cell carcinoma
IHC: immunohistochemistry
OPMD: oral potentially malignant disorder
OS: overall survival
OSCC: oral squamous cell carcinoma
PBS: phosphate-buffered saline
PD-1: programmed cell death receptor-1
PD-L1: programmed cell death ligand-1
STROBE: Strengthening the Reporting of Observational Studies in Epidemiology
